# Generation of Clonal Cultures of Adherent or Suspension Cells Using Flat Sessile Drops for Assurance of Monoclonality

**DOI:** 10.1002/bit.70030

**Published:** 2025-07-19

**Authors:** Joseph A. E. Morgan, Peter R. Cook, Alfonso A. Castrejón‐Pita, Edmond J. Walsh

**Affiliations:** ^1^ Department of Engineering Science University of Oxford Oxford UK; ^2^ Sir Willian Dunn School of Pathology University of Oxford Oxford UK

**Keywords:** cell‐line development, gene‐editing, microfluidics, sessile drop, single‐cell cloning

## Abstract

Single‐cell isolation is an essential step in many biomedical workflows, including genetic analyses and drug‐based assays. It is commonly attempted through limiting dilution into microtiter wells. However, dark optical edge effects at the well periphery make it difficult to confirm which wells contain just one cell. Consequently, statistical methods are used to obtain the probability that a well contains a single cell. Sessile microdrops can be deposited in the center of wells away from obscuring walls. If these drops have low contact angles, optical edge effects are minimal. A dilute cell suspension can be infused into such drops, which are then imaged to confirm the presence of a single cell with certainty. Subsequently, wells are flooded with media and incubated to allow clonal growth. The fraction of single cells yielding colonies then provides an accurate and non‐probabilistic measure of cloning efficiency. We demonstrate average cloning efficiencies between 62% and 78% with human embryonic kidney, cancer, and induced pluripotent stem cells, as well as Chinese‐hamster suspension cells. We verify that stem cells continue to express pluripotency markers after cloning and incorporate the method into a gene‐editing workflow for cell‐line development. This demonstrates the seamless integration of sessile microdrops into established protocols, providing assurance of monoclonality with high cloning efficiency.

## 1 Introduction

Single‐cell isolation and cloning are critical steps in many biomedical workflows such as drug development, cell‐line engineering, and gene‐editing (Chen et al. 2023). Isolation of a single cell can provide valuable information on gene regulation and biological mechanisms not identifiable from populations of cells (Heumos et al. 2023). The processes of single‐cell isolation and cloning present significant challenges. A common method involves limiting dilution into polystyrene microtiter plates: a stock solution is aliquoted into multiple wells such that each dispensed volume is likely to contain less than one cell (Fuller et al. 1988). This method relies on Poisson statistics and therefore users cannot be certain which aliquot contains just one cell. Consequently, multiple cloning rounds may be needed to provide confidence in monoclonality, adding significant time and cost (Soitu et al. 2020). In principle, monoclonality can be confirmed by imaging wells to verify the presence of a single cell post‐deposition. However, this is challenging due to optical edge effects at the junction between the well base and vertical wall that result in dark regions at the well periphery, where cells cannot be observed by conventional microscopy.

This problem has driven development of many sophisticated techniques. However, the utility, delicacy, and accessibility of these methods is often limited. For example, manual ones (such as micromanipulation) are typically low throughput and require skilled operators (Hu et al. 2016), while automated ones like fluorescence activated cell sorting (FACS) use high flow rates that can reduce cell viability (Hu et al. 2016). Furthermore, tagging with fluorescent markers to improve cell visibility can also reduce viability and/or may be inappropriate (Ge et al. 2013). Microfluidic approaches have emerged as promising alternatives (e.g., confining cells to individual droplets (Kang et al. 2014), hydrodynamic cell trapping (Zhou et al. 2021), imaging cells pre‐deposition), but their complexity, poor integration into existing workflows, and known reluctance of biologists to adopt microfluidics indicates there is still significant scope for innovation (Soitu et al. 2020; Whitesides 2006).

We present a simple and elegant approach: cells are imaged in sessile microdrops, that previous studies using an oil overlay have shown allow normal cell growth (Garcia‐Cordero and Fan 2017; Liberski et al. 2011; Prastowo et al. 2019; Soitu et al. 2018; Walsh et al. 2017). As sessile drops with high contact angles yield dark edge effects that are maximal at the drop periphery, we develop a workflow that involves imaging drops with reduced contact angles. We create such drops directly within microtiter wells, image them, and quickly fill the well with growth medium; this allows in‐well verification of monoclonality so that users can be certain which drops/wells contain only one cell (without requiring an oil overlay). Using this workflow, we show that various adherent and non‐adherent mammalian cells—including gene‐edited stem cells—clone efficiently, and that the method can be incorporated seamlessly into current cloning protocols involving microtiter plates.

## 2 Results

### 2.1 The Problem and a Solution

**Figure 1:**
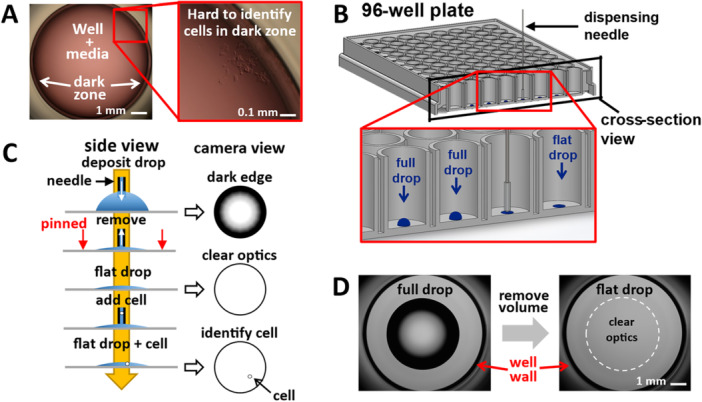
Identifying single cells in microtiter wells. (A) A microtiter well containing media viewed from beneath. Refraction of light at the junction between the well base and wall results a dark zone at the periphery where it can be difficult to identify cells. (B) A solution is to deposit a drop centrally within the well, away from the dark zone. Withdrawing volume from the drop leaves a flat drop. (C) Refraction of light across the curved surface of the original full drop results in the dark edges at the drop periphery, where it can be difficult to see drop contents. We exploit contact line pinning, whereby volume can be removed without a reduction in footprint diameter. This reduces the angle of incidence and eliminates dark regions within the sessile drop, giving a clear view of drop contents. Adding a small volume of fluid containing a single cell to the drop allows users to confirm the presence of a single cell microscopically. (D) Removing volume provides clear optics within the drop, where users can clearly identify cells. Depositing the drop centrally ensures the dark well periphery no longer compromises the image.

The most widely used method for cell cloning involves aliquoting a stock solution (where the dispensed volume typically contains < 1 cell) into wells in a microtiter plate (Fuller et al. 1988). Figure 1A is an image of a well filled with medium (200 µL) using this approach. When viewed from below, refraction of light at the plastic junction between the well base and wall yields a peripheral dark zone that obscures any cells, if present. Consequently, users can never be certain a well contains only one cell. Our approach involves depositing a microdrop (typically ~1 µL) in the center of a well (away from the dark peripheral zone), and we will see that refraction of light across the curved surface of such a drop yields a different kind of edge effect—this time at the drop periphery (Figure 1B,C). This second effect can also obscure cells; however, this edge effect can be minimized by reducing the angle of incidence at the drop periphery, which can be easily achieved by exploiting the phenomenon known as contact‐line pinning. Many cell‐culture media display strong pinning: volume can be removed from a drop without a reduction in footprint diameter (Soitu et al. 2020). This reduction in angle of incidence allows a clearer view throughout the drop (Figure 1C). We describe drops before and after removing volume as “full” and “flat.” A full drop yields strong edge effects of both types (Figure 1D, left), but the flat drop (90% volume removed) now has essentially no obscuring region at the drop periphery (Figure 1D, right).

### 2.2 Theory: Contact‐Line Pinning and Optics in Sessile Drops

**Figure 2:**
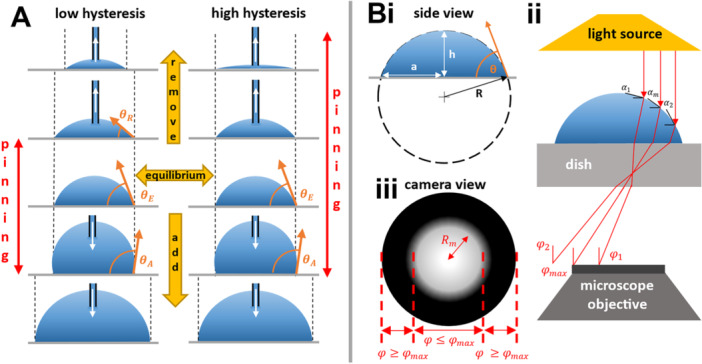
Contact‐line pinning and sessile‐drop optics. (A) Drops with low and high contact‐angle hysteresis. Middle row: drops deposited on a surface spread until the equilibrium contact angle, $\theta_{E}$, is reached. Two bottom rows: adding volume does not increase the footprint until the advancing contact angle, $\theta_{A}$, is reached. Two top rows: removing volume does not decrease the footprint until the receding contact angle, $\theta_{R}$, is reached. Drops are pinned between $\theta_{R}$ and $\theta_{A}$. Almost all fluid can be removed from a drop with high hysteresis without reducing footprint diameter. (B) Optical properties of sessile drops. (i) Geometry of spherical cap. (ii) Light from the microscope light source is refracted across the curved air‐drop interface, and light rays (red lines) beyond $\varphi_{\max}$ are not collected by the objective. (iii) In the camera view, lost light yields a dark zone (in which any cell present cannot be seen). Figure adapted from Soitu et al. (2020).

A drop deposited on a surface spreads to its equilibrium contact angle ($\theta_{E}$) which depends on multiple factors including interfacial tension, surface roughness, and temperature (Song and Fan 2021). Adding volume to a drop increases the contact angle, until the advancing contact angle ($\theta_{A}$) is reached and the drop footprint spreads. Conversely, removing volume reduces the contact angle, until the receding contact angle ($\theta_{R}$) is reached and the footprint retracts. The difference $\theta_{A}$ − $\theta_{R}$ is described as contact‐angle hysteresis and can be used to quantify contact‐line pinning—the degree to which the contact line is fixed (Figure 2A). Certain fluids are tightly pinned, such that > 95% volume can be removed without an observable reduction in footprint area ($\theta_{R}\;\ll$ $\theta_{E}$).

Maintaining low contact angles improves internal drop optics, since it minimizes the effects of the refraction of light across the curved interface. If a drop is sufficiently small (i.e., drop radius is below the capillary length, $\lambda_{c}$—which in water is ≈ 2.7 mm) where

$$\lambda_{c}=\sqrt{\frac{\gamma }{∆\rho g}},$$

and γ is surface tension, g gravitational acceleration, and ∆ρ the density difference between liquid and air, interfacial forces will outweigh gravitational ones, and the drop adopts a geometry that can be modeled using that of a spherical cap (Figure 2Bi). Drop volume, V, is therefore:

$$V=\frac{a^{3}}{\sin^{3}\theta }∙\frac{\pi \left(2-3\cos\theta +\cos^{3}\theta \right)}{3},$$

where θ is the contact angle and a the footprint radius. Maximum drop height, h, can be obtained easily:

$$h=\frac{a}{\sin\theta }\left(1-\cos\theta \right).$$

Knowing h and a, drop radius of curvature R is:

$$R=\frac{a^{2}+h^{2}}{2h}.$$

Drop curvature can then be used to predict how light is refracted across the curved liquid‐air interface. The critical angle $\varphi_{\max}$ beyond which light is refracted outside of the microscope objective is dependent on both numerical aperture NA and refractive index of the medium the light passes through, n:

$$\varphi_{\max}=\sin^{-1}\left(\frac{\mathrm{NA}}{n}\right).$$

If light is refracted beyond $\varphi_{\max}$ it will not be collected by the objective and this gives a dark region in the resulting image (Figure 2Bii,iii). The radius of the visible area within a drop ($R_{m}$) can be calculated using the radius of curvature and tangent between the drop surface and horizontal α using:

$$R_{m}=R\sin\left(\alpha_{m}\right).$$

The summation of refractions through each medium gives the total refraction, this includes through the drop ($n_{\mathrm{water}}\approx 1.33$), the polystyrene dish ($n_{\mathrm{dish}}\approx 1.5$), and air between dish and objective ($n_{\mathrm{air}}=1)$, determined using Snell's law:

$$n_{1}\sin\theta_{i}=n_{2}\sin\theta_{r},$$

where $\theta_{i}$ and $\theta_{r}$ are angles of incidence and refraction, and $n_{1,2}$ the refractive indices of the different materials (Soitu et al. 2020) (Supporting Information S1: Figure S1 shows experimental validation of theory).

### 2.3 The Workflow

**Figure 3:**
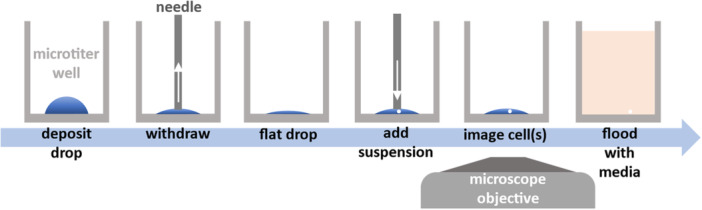
Using sessile microdrops in a cloning workflow. A drop is deposited into a microtiter well (typically 1 μL), volume withdrawn (90%) to leave a flat drop, and a small volume of cell suspension added (typically 50 nL). The now‐flat drop is imaged to confirm the presence of a single cell and flooded with media to allow normal cell growth.

Figure 3 outlines our workflow. A microdrop (typically 1 µL) is deposited in the middle of a conventional 96‐well microplate using a programmable 3‐axis traverse. Next, ~90% of the volume is removed to reduce the contact angle and produce a flat drop, a small aliquot of cell suspension added (typically 50 nL containing ~1.4 cells so that wells receive 0, 1, or 2 cells, calculated using Poisson statistics), the drop is imaged from below to confirm which wells contain just one cell, the well manually flooded with growth medium (typically 200 µL), and the microplate incubated to allow clonal growth. Finally, cloning efficiencies are determined. Conventional limiting dilution is used as a control; however, results from this control are subject to substantial uncertainties associated with counting and diluting cell numbers accurately. Our workflow depends on two critical steps: ensuring pinning lines do not retract when fluid is withdrawn to create flat drops, and that these drops are sufficiently flat to lack obscuring dark zones at their periphery. If there is no hysteresis, the pinning line will retract as fluid is removed to maintain a constant equilibrium contact angle and hence retain dark zones.

### 2.4 Pinning‐Line Stability

Fetal bovine serum (FBS) is present in most media used to culture mammalian cells; it also stabilizes pinning lines (Soitu et al. 2020). Unfortunately, it is a complex, undefined, and variable bioproduct that contains many different proteins—and this is driving development of chemically‐defined alternatives (Gstraunthaler et al. 2013). As we wished to develop an alternative that provides excellent pinning on the different polystyrene surfaces found in the microplates used to culture adherent and nonadherent cells, we screened various fluids (Supporting Information S1: Figure S2). Phosphate‐buffered saline (PBS) is perhaps the simplest and most widely used salt solution in cell culture, and when supplemented with 0.5 mg/mL bovine serum albumin (BSA)—but not a non‐protein alternative like poly‐ethylene glycol (PEG)—it pinned micro‐drops on both types of surface (Supporting Information S1: Figure S2). Therefore, generally we use it when creating flat drops.

### 2.5 Optical Properties of Flat Drops

**Figure 4:**
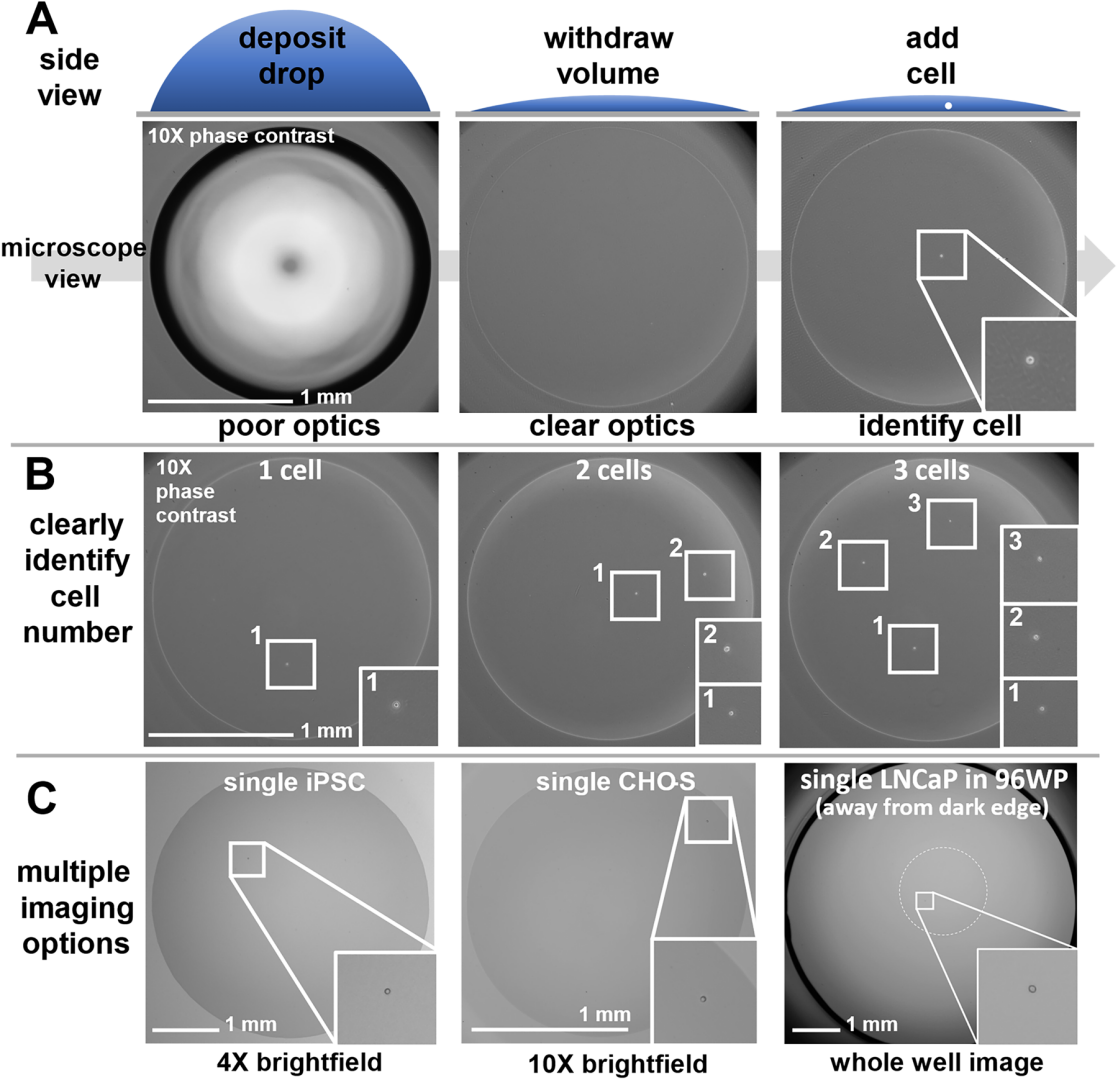
Identify cells in microdrops. (A) Method for identifying a single cell. A drop initially has poor optics, withdrawing volume improves optics and—after adding a single cell—the cell can be seen clearly. (B) Users can count numbers of cells (here, from human embryonic kidney) per well accurately. (C) Cells can be seen clearly using 4x and 10x objectives, in brightfield or phase contrast. The dotted circular white line marks the drop periphery in the whole‐well images (where edge effects at the well periphery are clearly visible).

Figure 4A shows a 1 μL drop of PBS + 0.5 mg/mL BSA deposited on a polystyrene surface treated for tissue culture (see Section 4 for details). The drop has a high contact angle ($\theta_{E}$ ≈ 60°), and a dark zone at the drop periphery is clearly seen in the microscope when viewed from below. Removing 0.9 μL does not reduce footprint diameter; the drop is pinned (*θ* ≈ 7.3°) and now optics across the entire drop footprint have become much clearer. After adding 0.05 μL containing ~1.4 cells users can now count precisely the number of cells delivered (here, one) despite the (slight) increase in contact angle (*θ* ≈ 11°) because the total refraction is still below the critical angle (14.5° for the 10x objective).

Drops with different numbers of cells can be imaged clearly (Figure 4B) using a standard microscope. Figure 4C shows that a variety of cell types are clearly identifiable using 4x and 10x objectives. Here, we show a single (adherent) induced pluripotent stem cell (iPSC) in a flat drop (5 µL PBS + 8 µg/mL laminin initially deposited, 90% volume removed), a single nonadherent CHO‐S cell in a flat drop (1 µL PBS + 0.5 mg/mL BSA initially deposited, 90% removed), and a whole‐well view of a single adherent LNCaP cell in a flat drop (same as for CHO‐S). In all cases, images of single cells are no longer compromised by edge effects where the well base meets the wall, or by curvature at the drop periphery.

### 2.6 Cloning Efficiencies

**Figure 5:**
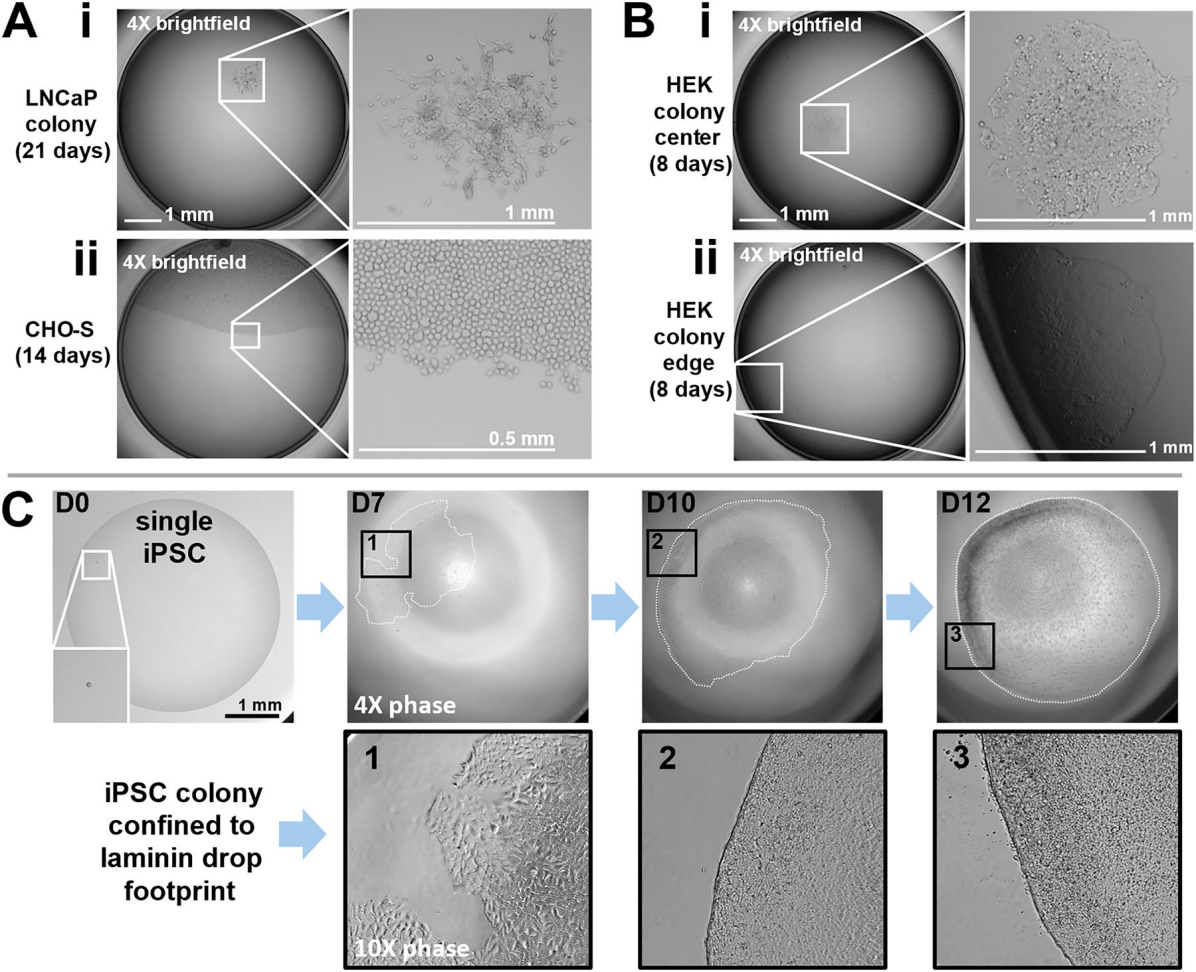
Single cells grow to colonies. (A) Whole‐well images of colonies of (i) LNCaP cancer cells (d 21), and (ii) CHO‐S (d 14). (B) Whole‐well images of colonies growing in (i) the center of a well or (ii) at the well periphery, where they are partly obscured from view. (C) Part‐well images of an induced pluripotent stem cell (iPSC) (d 0) growing to a colony (d 7, d 10, d 12; dashed white lines outline colony, magnifications of boxed regions 1–3 shown below, image contrast enhanced and sharpened). Colony is confined to the footprint of initial laminin coating drop, providing a clear whole‐colony image.

Figure 5A shows example clonal colonies of LNCaPs after 21 days of incubation, and CHO‐S after 14, both of which proliferated from a single cell. As expected using this method, adherent colonies usually grow in the middle of the well (Figure 5Bi) in which case the entire colony can be seen clearly, away from the dark well edges. However, some grow in the dark zone at the well periphery (Figure 5Bii); this highlights the need for confidence that a well initially contained only one cell.

A range of matrix coatings were tested to assess which cell‐culture fluids are tightly pinned to polystyrene surfaces (Supporting Information S1: Figure S1). For example, laminin is commonly used in the culture of iPSCs. After 7 days, colonies growing from a single iPSC can be clearly identified growing on the matrix (Figure 5C). Such colonies grow to fill the original footprint of the drop, without extending into the noncoated region of the well that is now covered with medium only. In other words, iPSCs do not grow beyond the edge of the laminin coating, giving a clear image of the whole colony in the center of the well. Here, larger 5 µL drops were used, providing a larger footprint area permitting larger colony growth before passaging.

**Figure 6:**
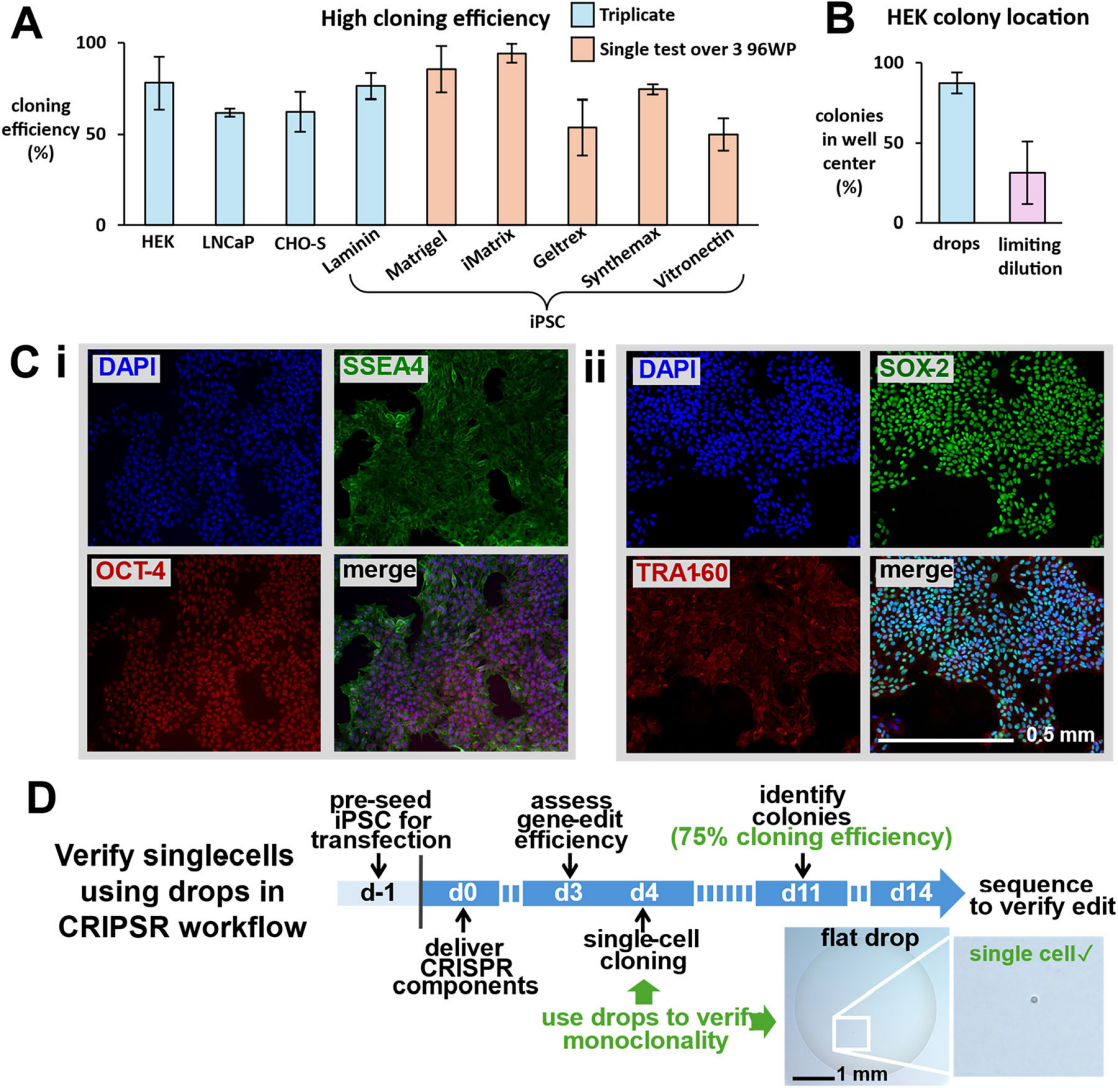
Cloning efficiencies, colony characteristics, and a CRISPR workflow. (A) Excellent cloning efficiencies are obtained after imaging in drops (error bars ± standard deviation). (B) HEK colonies are more likely to grow in the center of the well after imaging in microdrops. (C) Induced pluripotent stem cell (iPSC) pluripotency is retained postimaging, demonstrated by (i) markers for SSEA‐4/OCT‐4 and (ii) SOX‐2/TRA1‐60. (D) Timeline demonstrating how single‐cell verification using flat sessile drops is incorporated into a CRISPR gene‐edit workflow.

Figure 6A shows some cloning efficiencies achieved using various cells, growth media, and coatings: all are in the expected range and validate the gentle handling of single cells throughout the workflow. Each triplicate represents average cloning efficiencies across 3 × 96‐well plates (three biological reps). iPSC cloning efficiencies on various matrix coatings represent a single biological rep averaged over 3 × 96‐well plates (apart from laminin). We also noticed that (adherent) human embryonic kidney (HEK) colonies often grew closer to the center of the well compared to those formed using the conventional method (~87% in the center, vs. ~31% respectively, Figure 6B). This is likely attributed to the initial central deposition in the well—a cell falls to the bottom and then is not displaced when the well is flooded with medium. Conversely, during conventional cloning, cells are prone to settle at the well periphery. As cell stress and changes in microenvironment can impact differentiation of iPSCs (Zhang et al. 2022), we performed quality‐control tests which showed that various pluripotency markers (i.e., SSEA‐4, OCT‐4, SOX‐2, and TRA1‐60) continued to be expressed normally after cloning (Figure 6C, see Supporting Information S1: Figure S4 for conservation using coatings other than laminin, plus staining for SSEA‐1, which we use as a negative control).

### 2.7 Incorporation Into a Gene‐Editing Workflow

Finally, we integrated our method into a CRISPR gene‐editing workflow (Figure 6D; Supporting Information S1: Figure S5 provides detail). Clonal selection is an essential step in such workflows as not all cells are edited. The MYBPC3 gene (which encodes cardiac myosin building protein) is important in cardiac pathophysiology (Carrier et al. 2015), and we knocked it out (through frameshift mutations, Lalonde et al. 2017) in human iPSCs using a standard protocol (Ludwik et al. 2023). Three days after editing, samples were sequenced and showed 75% knockout efficiency—a measure of how many of the contributing indels are likely to result in a functional knockout (Bryant 2018). On Day 4 (d4), a dilute solution of edited cells was dispensed into flat drops of PBS plus 8 µg/mL laminin in microtiter wells; 61 drops contained single cells, and—after flooding with medium, 46 of these single cells grew to give colonies by d14 (75% cloning efficiency). Twelve of these colonies were now randomly selected, the targeted segment amplified, and sequences determined. Eleven of the twelve clones had been edited. This provides a proof‐of‐concept that our method can be incorporated seamlessly into standard editing protocols for cell‐line development.

## 3 Discussion

Poor optics at the edge of microtiter wells make single‐cell verification challenging. Depositing cells within a sessile drop in the center of a well and so away from the edge mitigates against this. However, it introduces another problem: drops with high contact angles exhibit poor optics at their periphery due to refraction across the air–liquid interface. We leverage contact‐line pinning to reduce the contact angle; this improves optics and enables visual confirmation of monoclonality within microtiter wells (Figure 1).

We screened various fluids to see which are pinned to polystyrene surfaces (Figure 2) and found that some protein‐rich solutions—including FBS—are successfully pinned (Supporting Information S1: Figure S2A–C). This aligns with previous studies (Prastowo et al. 2019; Soitu et al. 2020) and is likely due to protein adsorption at the pinning line (Berejnov 2008). However, FBS is an ill‐defined and variable bioproduct (Gstraunthaler et al. 2013), and it's critical constituent—albumin (from bovine or human serum (Jayme and Smith 2000), Supporting Information S1: Figure S3)—pinned just as well at concentrations one‐tenth that found in human serum (~50 mg/mL) (Taverna et al. 2013). Using a three‐axis traverse to automate the workflow (Figure 3), microdrops were deposited, 90% drop volume withdrawn, a small volume of cell suspension added to yield drops free of edge effects and drops imaged (using 4x and 10x plus brightfield or phase contrast). Wells containing single cells were identified (Figure 4) and quickly filled with medium (peripheral sacrificial wells were filled with PBS to minimize the effects of evaporation on cell survival in the inner ones). Automated seeding of cells into drops took ~1 s per well. Our total workflow took ~20 min to process all 60 inner wells (~5 min per 15 wells) of a 96‐well plate. Single cells were in drops for no more than ~2 min (imaging time) before flooding with media. We calculated that an integrated liquid handling and imaging system would take ~5 min to process an entire 96‐well plate from seeding of single‐cells to flooding wells with media. For iPSCs, larger drops (5 µL; ~3.8 mm diameter) were deposited to create a larger footprint and permit greater cell proliferation on the matrix‐covered area. Here, withdrawing 90% volume and adding 0.5 µL cell suspension gives an imaging contact angle of ~10.6° (laminin drop).

Colonies are clearly identified using a conventional microscope on d7 for rapidly growing iPSCs, d8 for HEKs, d14 for CHO‐S, and d21 for slow‐growing LNCaP (Figure 5). Cloning efficiencies are equivalent to those seen conventionally using limiting dilution, including those for iPSCs grown on a wide range of matrices without loss of pluripotency markers (Figure 6A,C; Supporting Information S1: Figure S4). Additionally, our approach is easily incorporated into an established gene‐editing protocol (Figure 6D).

Our workflow has various advantages, with the most important being the certainty that a colony is derived from a single cell. Furthermore, coating just the area of microdrop footprint compared to the whole well provides savings in expensive coating reagents (e.g., coating a drop footprint area of 8 mm^2^ gives a 75% reduction in coating consumption compared to traditional methods in 96‐well plates; Supporting Information S1: Figure S2D). Adding cells to microdrops centrally in wells also provides some control over where a cell first adheres to the surface, and so ultimate colony location (e.g., adherent HEKs are usually not dislodged during well flooding, and this allows a clear whole‐colony view in the center of the well, Figure 5B). This facilitates earlier colony identification, earlier passaging, and so reduced experiment time compared to the conventional approach.

Our method has limitations. First, infusion of cells into drop footprints is governed by Poisson statistics—users cannot guarantee they will get a single cell per drop. Some wells will receive no cells (others more than one), and visual confirmation is required to determine which contain just one cell. However, the number of wells receiving one cell could be improved by re‐infusing empty drops with an additional aliquot. Second, counting errors commonly lead to infusion of too many (or too few) cells in a drop; however, as users can count exact cell numbers in each drop, they can work backwards to determine the true concentration dispensed. For example, we use a simple maximum likelihood estimation to fit Poisson distributions to iPSC cell counts per well (18 × 96‐well plates in total) and find that on average, 1.87 cells were deposited per well (standard deviation of ±0.44); this indicates that the true cell concentration was higher than counted manually (target of 1.4 cells per well, Supporting Information S1: Figure S6)**.** Note that limiting dilution experiments are subject to the same uncertainty, with some experiments yielding (apparent) cloning efficiencies of > 100%, or misleadingly poor ones. Third, drop footprint diameter is limited by well diameter (6 mm; then, for example, using a 0.5 mm tolerance, the maximum possible footprint diameter is 5 mm—that of a ~20 µL drop on treated polystyrene. Finally, drops must be positioned away from the well walls to avoid well edge effects.

In summary, flat sessile drops provide excellent optics for imaging single cells in standard microwells, and cells clone efficiently after imaging. This provides certainty in monoclonality, and the method can be incorporated seamlessly into existing workflows.

## 4 Materials and Methods

### 4.1 Cells

Cells were grown routinely at 37°C and 5% CO_2_, counted manually using a haemocytometer unless stated otherwise, and diluted to the required concentration.

HEK 293 cells (Russell et al. 1977) were cultured in 5 mL DMEM (Sigma‐Aldrich) + 10% FBS (Gibco) + 1% Penicillin‐Streptomycin (Gibco) in 25 cm^2^ polystyrene canted neck flasks (Corning). They were prepared for experiments by removing medium, rinsing with 1 mL PBS (Sigma‐Aldrich), detached from the polystyrene surface (1 mL trypsin; TrypLE Express, Gibco), incubated (37°C, 2 min), trypsin was neutralized by adding 4 mL media, cells transferred to a 15 mL centrifuge tube (Falcon), (500 rpm; 3 min), the supernatant removed, cells resuspended in PBS and counted.

Lymph node carcinoma of the prostate (LNCaP) cells (Horoszewicz et al. 1983) were cultured in 20 mL RPMI‐1640 medium (Sigma‐Aldrich) + 10% FBS + 1% MEM nonessential amino acids (Gibco) + 1% penicillin‐streptomycin + 1% L‐Glutamine (Gibco) + 1% sodium pyruvate (Gibco) + 1% MEM vitamin solution (Gibco) in 75 cm^2^ U‐shaped canted neck cell culture flasks with vent cap (Corning). Cells were prepared for experiments as above with the following variations: rinse with 2.5 mL PBS, detach with 3 mL trypsin, 4 min incubation, neutralize with 4 mL complete medium, and spin for 5 min.

Chinese hamster ovary suspension (CHO‐S) cells (Kito et al. 2002) were cultured in CD CHO medium (Gibco) + 40 mL/l L‐Glutamine in 25 cm^2^ polystyrene canted neck flasks stood vertically to inhibit cell attachment. When cloning, nontreated (suspension) microtiter wells were flooded with a 1:1 ratio of CD CHO + 40 mL/l L‐glutamine and DMEM/F12 (1:1) (Gibco) since this combination is known to support clonal growth of CHO‐S (Zhu et al. 2012).

KOLF2‐C1 iPSCs (Pantazis et al. 2022) were cultured in StemFlex + supplement (Gibco) + 1X CloneR2 (Stemcell Technologies) in 6‐well clear tissue‐culture‐treated plates (CytoOne, Starlab) coated with Biolaminin 521 (BioLamina) as per manufacturer's instructions. Cells were prepared for experiments by rinsing with 1 mL PBS, detach with 1 mL trypsin, 5 min incubation, neutralize with 2 mL StemFlex + supplement + CloneR2 per well. Cells were spun (300 g; 5 min), supernatant discarded, resuspended in 1 mL complete media, and stained using Trypan Blue (Gibco) for counting.

Cells suspensions were aspirated manually via pipette before collection by the dispensing system, mitigating sedimentation within the reservoir.

### 4.2 Drop Optics Theory Validation

To validate the critical‐angle theory for drops, we dispensed a 1 μL drop of PBS + 0.5 mg/mL BSA onto a 60 mm treated polystyrene dish (Corning) using a syringe pump (Harvard Apparatus) and 50 μL syringe (Hamilton). Sacrificial drops (~10 μL) were manually deposited around the central drop to mitigate evaporation. The drop was subsequently imaged from below, and footprint measured using ImageJ (Schneider et al. 2012).

Volume was incrementally removed from the drop in 100 nL steps (10% of the initial volume) using the syringe pump, imaging after each withdrawal. The radius of the visible area was determined by vertical lines through the drop center and plotting corresponding gray values; the boundary for determination of critical angle was defined when there was a 15% decrease in pixel intensity observed compared to the center of the drop. Measurements were averaged along the vertical line and compared with the analytical results presented in Supporting Information S1: Figure S1.

### 4.3 Screening Fluids Used to Create Drops for Cell Imaging

To assess the ability of a fluid to retain a low contact angle, 5 μL drops were deposited manually onto a 60 mm polystyrene tissue‐culture‐treated dish (Corning) and the footprint imaged. Approximately 4.75 μL (95% drop volume) was then manually removed from the drop via pipette and footprints re‐imaged. If there was no visible change in drop footprint diameter, the fluid can be considered sufficiently pinned to facilitate imaging. For each fluid this process was repeated on nontreated 60 mm polystyrene dishes (Corning). Fluids tested include deionized water, PBS, DMEM, a range of polyethylene glycol solutions (PEG, Sigma‐Aldrich), and solutions containing 0.05%, 0.5%, and 5% BSA. All solutions were prepared in PBS unless stated otherwise.

Matrix coatings for iPSCs (5 μL) were deposited into each well of a 96‐well tissue‐culture‐treated microtiter plate (Corning). Drops were incubated for the manufacturer's recommended duration (typically 1–2 h), imaged using an inverted microscope (Olympus IX53), 4.5 μL removed, and drop footprints re‐imaged. Drops were then measured using FIJI (imageJ, Schneider et al. 2012) and footprint diameter compared before and after removing volume to determine whether the fluid is pinned. Various matrix coatings were tested including human recombinant laminin 521 (LN521, BioLamina), Matrigel hESC‐Qualified Matrix (Corning), iMatrix‐511 (TakaraBio), Geltrex LDEV‐Free, hESC‐Qualified reduced growth factor basement membrane matrix (Gibco), Vitronectin recombinant human protein (Gibco), and Synthemax II‐SC substrate (Corning). Concentrations used in creating drop footprints can be found in Supporting Information S1: Figure S2D.

### 4.4 Automated Drop Deposition

The deposition, addition, and withdrawal of fluid from drops was automated using a programmable motorized three‐axis traverse and incorporated syringe pump (iotaSciences). This was used to give precise spatial control of a 25‐gauge dispensing needle connected to a 500 μL glass syringe (Hamilton) via 28‐gauge PTFE tubing (Adtech). All fluids used in drop deposition were aliquoted into 1.5 mL microtubes (Eppendorf) for handling by the printer. Printer commands were written in GCMC and compiled to G‐code. The tubing, needle, and syringe were kept filled with 70% ethanol solution when not in use. Drops for imaging iPSCs (matrix coatings) were deposited using a similar three‐axis traverse with incorporated syringe pump (isoPick, iotaSciences) in which fluid was handled via a 23‐gauge dispensing needle (Tomlinson Tube) connected to a 500 μL glass syringe (Hamilton) via 24‐gauge PTFE tubing (Adtech).

### 4.5 Drops for Imaging HEK, LNCaP and CHO‐S

Drops (1 μL PBS + 0.5 mg/mL BSA) were deposited into the central 60 wells of a 96‐well microtiter plate (peripheral wells were prefilled with 200 μL PBS to reduce evaporation in central wells). For HEK and LNCaP, drops were deposited in a flat bottom tissue‐culture‐treated 96‐well plate, and for CHO‐S in a flat bottom nontreated 96‐well plate (both Corning). Fluid to create drops was collected by the printer and separated from ethanol within the tubing by a PBS slug (10 μL) flanked by two air gaps (3 μL). Drops were deposited with the tip of the dispensing needle 0.5 mm above the well base. This process was done 15 wells at a time to reduce the time a drop was left exposed to air. Next, 0.9 μL was withdrawn from each drop, by inserting the dispensing needle into the drop at a height of 0.1 mm from the well base. Next, 0.05 μL cell suspension (~28,000 cells/mL) was infused into the now‐flat drop from a height of 0.1 mm from the well base. The plate was then transferred from the printer to the microscope for imaging. Finally, wells were flooded manually with 200 μL media before starting the process again for the next block of 15 wells.

### 4.6 Drops for Imaging IPSCs

Matrix coatings (5 μL) was deposited (from a height of 1 mm from the well base) into the central 60 wells of a 96‐well microtiter plate. Drops were then incubated (37°C, 5% CO_2_) for the time recommended by the manufacturer (1 h for Matrigel, Geltrex, Synthemax, and vitronectin. 2 h for laminin; no incubation required for iMatrix). After incubation, 4.5 μL was withdrawn from drops to reduce the contact angle. This was done 15 drops at a time to reduce the time drops were exposed to air. Next, 0.5 μL cell suspension was added to drop footprints (2800 cells/mL, at a height of 0.15 mm from the well base). The plate was then transferred from the printer to the microscope for imaging. Finally, wells were flooded manually with 100 μL media (reduced volume to save comparatively expensive culture media for iPSCs). Wells containing a single cell were manually topped up with an additional 100 μL media after 5 days to maintain healthy colony growth.

### 4.7 Limiting‐Dilution Control Experiments

In each case, control experiments were performed using limiting dilution into 96‐well microtiter plates. Stock concentrations were diluted to 2.5 cell/mL in 14 mL media in a 15 mL centrifuge tube (Greiner). From this suspension, 200 μL was manually deposited into each of the inner 60 wells of a 96‐well microtiter plate. In the case of iPSCs, the same concentration was achieved in 7 mL media, and 100 μL was manually deposited into each inner well to reduce consumption of expensive media.

### 4.8 Cloning Efficiency in Microtiter Plates

Cloning efficiency is typically calculated using:

$$\text{Cloning efficiency}\left(\%\right)=\left(\frac{\text{colonies from single cell}}{\text{single cells}}\right)\times 100.$$

However, for the reasons previously discussed, the true number of single cells actually present in a microtiter plate is often unknown. This means that users rely on Poisson statistics to give the probability P(x) that a well contains a single cell:

$$P\left(x\right)=\frac{e^{-\lambda }\lambda^{x}}{x!},$$

where x is the number of cells (1 for a single cell), and *λ* the average number of cells per well (typically ≪ 1). Using this method, users can estimate the number of wells expected to contain a single cell for a given stock concentration. This measure of cloning efficiency is a probability‐based estimate and depends on an accurate estimate of cell concentration. Errors in cell counting, which are common with manual counting, can lead to cloning efficiencies even greater than 100%. Similarly, low cloning efficiencies might misleadingly indicate poor cell viability when they could be a consequence of counting error.

### 4.9 Markers for iPSC Pluripotency

Single cells were deposited into flat drops of laminin, Matrigel, and iMatrix coatings within microtiter wells (96‐well plate) on d0. On d3, media was changed (StemFlex + CloneR2) and on d5, colonies from single cells identified and media changed to StemFlex only. On d7, colonies were transferred from respective coatings and split across three wells of a LN521‐coated 6‐well plate. On d14, cells were frozen in Knock‐out serum replacement (Gibco) + 5% DMSO (Sigma) at 1 × 10^6^ cells/mL in 1.5 mL vials (Nalgene, ThermoFisher). Vials were then sent to Cellected (Cellected Ltd, Salisbury, UK) for testing and analysis of pluripotency; five vials contained cells grown on laminin, five grown in iMatrix, four grown on Matrigel, and one parental (not cloned) grown on laminin which we use as a control.

After recovery of frozen cells in StemFlex media on laminin matrix, cells underwent morphological assessment and immunocytochemistry/immunofluorescence (ICC/IF) tests for Oct4, SSEA4, Sox2, Tra‐1‐60 (positive control) and SSEA1 (negative control) following ISCCR guidelines (Basic Research Standards 2023); all gave results as expected. The commercial service also included analysis using a single‐nucleotide polymorphism (SNP) array (Infinium GSA v3), plus tests for mycoplasma and sterility; results were as expected and not shown.

### 4.10 CRISPR Gene‐Edit Workflow

Flat drops were incorporated into a CRISPR gene‐editing workflow in collaboration with iotaSciences following the protocol in Ludwik et al. (2023) On d1, KOLF2‐C1 were suspended in StemFlex + 1X CloneR2 and plated (20,000 cells/well) into two wells of a tissue‐culture‐treated six‐well plate that had been precoated with Biolaminin 521, and incubated overnight. On d0, transfection was carried out using the Neon Nucleofector and Neon Transfection system 10 µL kit (Invitrogen). RNP comprised 2 µL MYBPC3 G > GG gRNA that targets exon 24 (target sequence AGGACTCCTGCACAGTACAG), 0.5 µL Cas9 (both Integrated DNA Technologies) and 12.5 µL Buffer R (ThermoFisher). Cells were harvested from one well of a six‐well plate, transferred to a microcentrifuge tube and resuspended in 10 µL Buffer R. Cells were then electroporated, and plated in a six‐well plate coated with Biolaminin 521 in StemFlex + 0.5X CloneR2. Subcultured wild‐type (control) cells were plated in an additional well and media replaced daily.

To assess the edit efficiency in the pool of transfected cells, a whole‐cell lysate was prepared on d3. Media was removed and 25 µL lysis buffer (made by adding 2.5 µL DNARelease to 100 µL dilution buffer—both from ThermoFisher) added per well, then incubated (2 min) at room temperature. Cell lysate was collected and transferred to a PCR tube, incubated (98°C, 2 min), spun down, and the supernatant stored at −20°C. PCR was then carried out using Phire Tissue Direct PCR MasterMix kit (ThermoFisher) and a MiniAmp Thermal Cycler (Applied Biosystems) using MYBPC3 G > GG forward (sequence CAGCCTGTGGCGGTTAGTT) and reverse (sequence CGCTTCATGACTCAGCTCCT) primers, following the manufacturer's protocol (Thermo Scientific Phire Tissue Direct PCR Master Mix Product Information 2015). Purified human genomic DNA (Promega) and water were used as positive and negative controls. PCR products were purified using the GeneJET PCR Purification Kit (ThermoFisher) following the manufacturer's protocol (Thermo Scientific GeneJET PCR Purification Kit User Guide 2015). Finally, 5 µL of the purified PCR product + 5 µL forward primer MYBPC3 G > GG (10 µM) were sent to Source Biosciences (Source Bioscience Limited, Nottingham, UK) for Sanger sequencing and determination of knockout efficiency in the pool using the ICE (inference of CRISPR edits) tool (Conant et al. 2022).

On d4, 5 µL drops (Biolaminin 521) were deposited into the inner wells of a tissue‐culture‐treated 96‐well plate, incubated (2 h), and volume withdrawn as done in previous cloning tests. A suspension of the edited cell pool (2 cells/µl) was prepared in StemFlex + 1X CloneR2, and 0.5 µL suspension added to flat drops, which were then imaged and wells flooded with media. Wells containing a single cell were identified, and media changed daily thereafter. On d11, colonies were identified and cloning efficiency calculated. On d14, 12 of the clones were selected and target regions in each amplified using the sequencing primer (as on d3) and sequenced (Source Biosciences). Sequences obtained are shown in Supporting Information S1: Figure S5.

### 4.11 Imaging

Drops containing HEK, LNCaP, and CHO‐S and subsequent colonies were imaged using a standard inverted microscope (Olympus IX53) and coupled DSLR camera (Nikon D610). Drops containing iPSCs and subsequent colonies were imaged using an inverted microscope (isoHub, iotaSciences) and attached camera (MC203CG‐SY‐UN, xiC, ximea). Image processing and analysis was done in FIJI (imageJ) (Schneider et al. 2012).

### 4.12 Calculations and Statistical Analysis

All calculations (e.g., drop geometry, effect on adding/removing drop volume on contact angle) and statistical analysis (e.g., maximum likelihood estimation for fitting Poisson distribution to data) were performed in either Excel (Version 2404, Microsoft) or MATLAB (2023a, Mathworks).

## Author Contributions

J.A.E.M., P.R.C., and E.J.W. designed research. J.A.E.M. performed research. J.A.E.M., P.R.C., A.A.C., and E.J.W. analyzed data, wrote and reviewed the paper.

## Conflicts of Interest

Edmond J. Walsh is an inventor on a patent family related to the subject of this paper. The remaining authors declare no conflicts of interest.

## Supporting information

Bio tech eng Cloning paper SI.

## Data Availability

The data that support the findings of this study are available from the corresponding author upon reasonable request.
