# Supplementary material for: Generation of Clonal Cultures of Adherent or Suspension Cells Using Flat Sessile Drops for Assurance of Monoclonality
Source: Biotechnol Bioeng. 2025 Jul 19;122(10):2739–50. doi: 10.1002/bit.70030 (PMC12417753; doi:10.1002/bit.70030)
Supplement: Supplementary file 1 — Bio tech eng Cloning paper SI. [file BIT-122-2739-s001.docx]

**Supporting Information for**

Generation of clonal cultures of adherent or suspension cells using flat sessile drops for assurance of monoclonality

Joseph A. E. Morgan^1^, Peter R. Cook^2^, Alfonso A. Castrejón-Pita^1^, Edmond J. Walsh^1*^

^1^Department of Engineering Science, University of Oxford, Oxford, OX1 3PJ, UK

^2^Sir Willian Dunn School of Pathology, University of Oxford, Oxford, OX1 3RE, UK

*Corresponding author: Edmond J. Walsh

**Email:**  [edmond.walsh@eng.ox.ac.uk](mailto:edmond.walsh@eng.ox.ac.uk)

**This MS Word file includes:**

Figures S1 to S6

Supporting Information


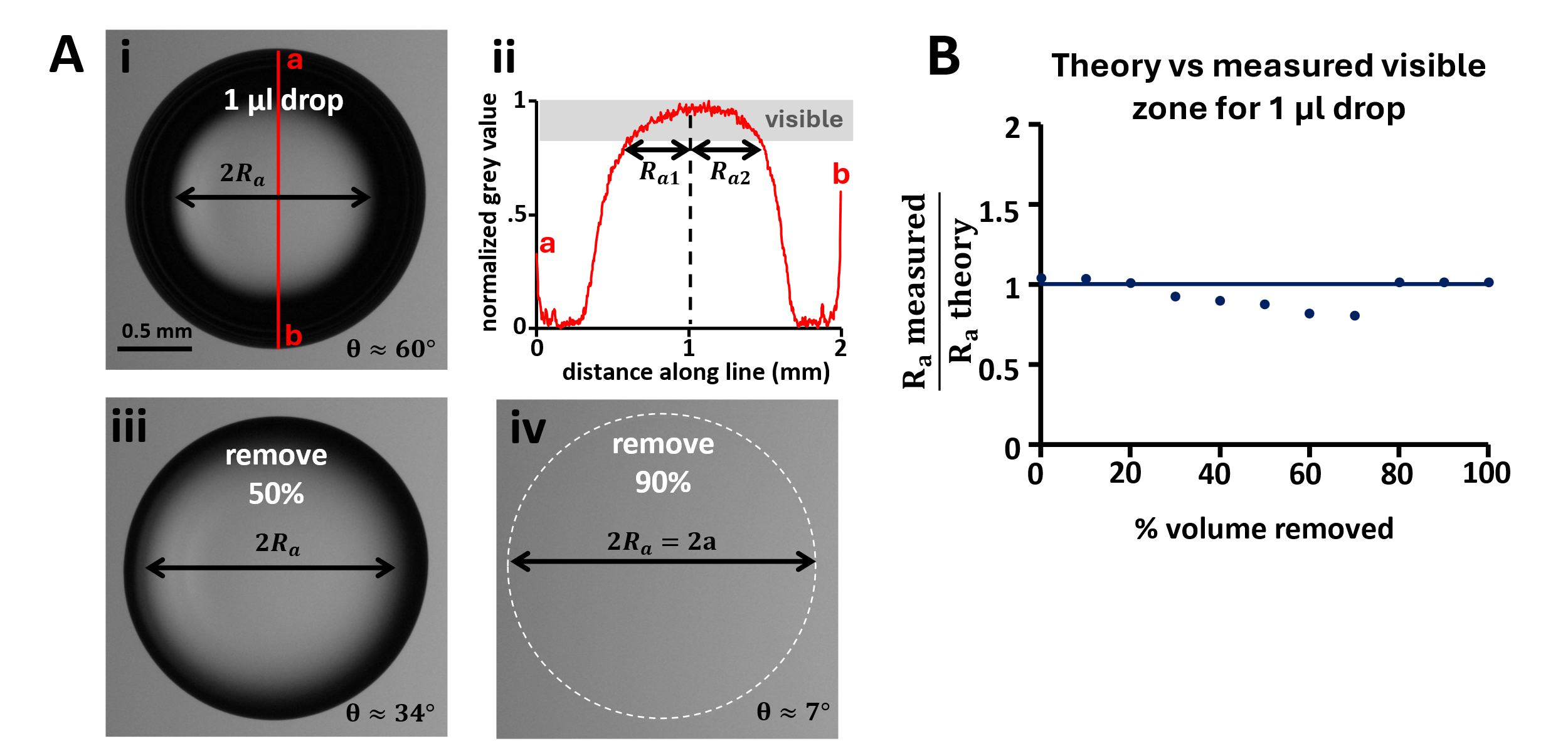


Fig. S1. Drop optics theory validation.

**(A)** A 1 μl drop of PBS + 0.5 mg/ml BSA is deposited on a dish (**i**); it has dark (optically inaccessible regions at the drop periphery). The radius of the visible area (Ra) is determined by plotting the grey values along a vertical line through the drop center (**ii**); the visible boundary was defined as a 15% decrease in pixel intensity compared to the center of the drop. Removing 50% of the volume (**iii**) reduces the angle of incidence and therefore total refraction, shrinking the dark zone. Removing 90% of the volume (**iv**) gives clear optics across the entire footprint (pinning line highlighted, images taken in brightfield using 4X objective).

**(B)** Comparison between the predicted radius of visible area and experimental measurement supports the theory with reasonable accuracy.

**
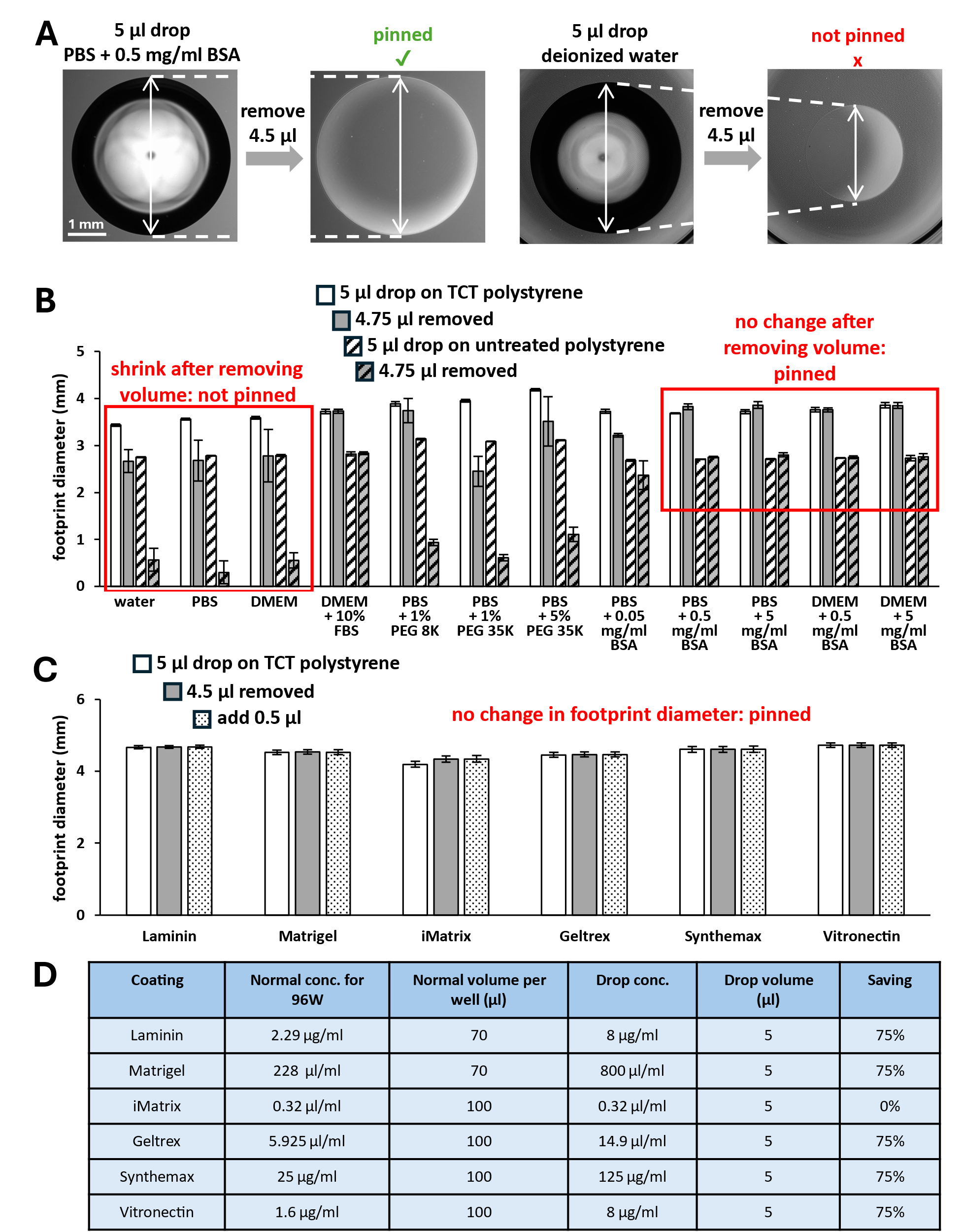
**

**Fig. S2. Drop footprint analysis.**

**(A)** A drop on tissue-culture-treated polystyrene imaged from below. Removing volume from a drop of laminin does not cause a reduction in footprint diameter; the footprint remains pinned at the original position. Removing volume from a drop of deionized water causes a clear reduction in footprint diameter (as the pinning line retracts).

**(B)** Fluids were evaluated to determine whether they are pinned on both treated and untreated polystyrene.

**(C)** Matrix coatings were assessed for any change in footprint diameter after removing and adding volume.


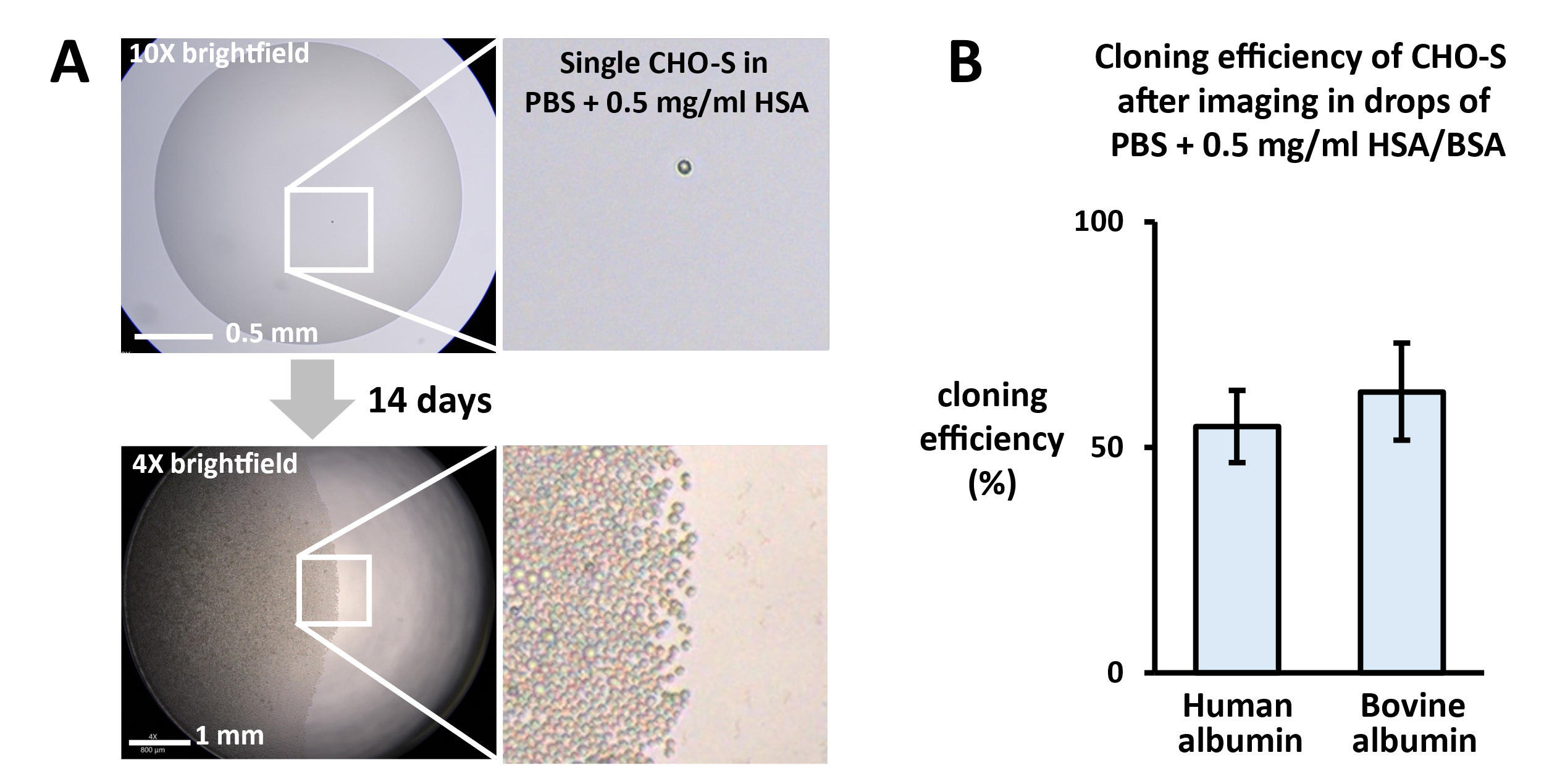


Fig. S3. Imaging CHO-S in drops containing bovine vs human serum albumin (HSA)

**(A**) Drops of PBS + 0.5 mg/ml HSA are pinned on polystyrene surfaces, allowing creation of drops with low contact angles and excellent optics required for single-cell imaging. A single CHO-S is clearly identified and grows into a colony in a non-treated (suspension) microtiter well.

**(B)** There is no significant difference in cloning efficiency after imaging CHO-S in drops containing HSA vs BSA.

**
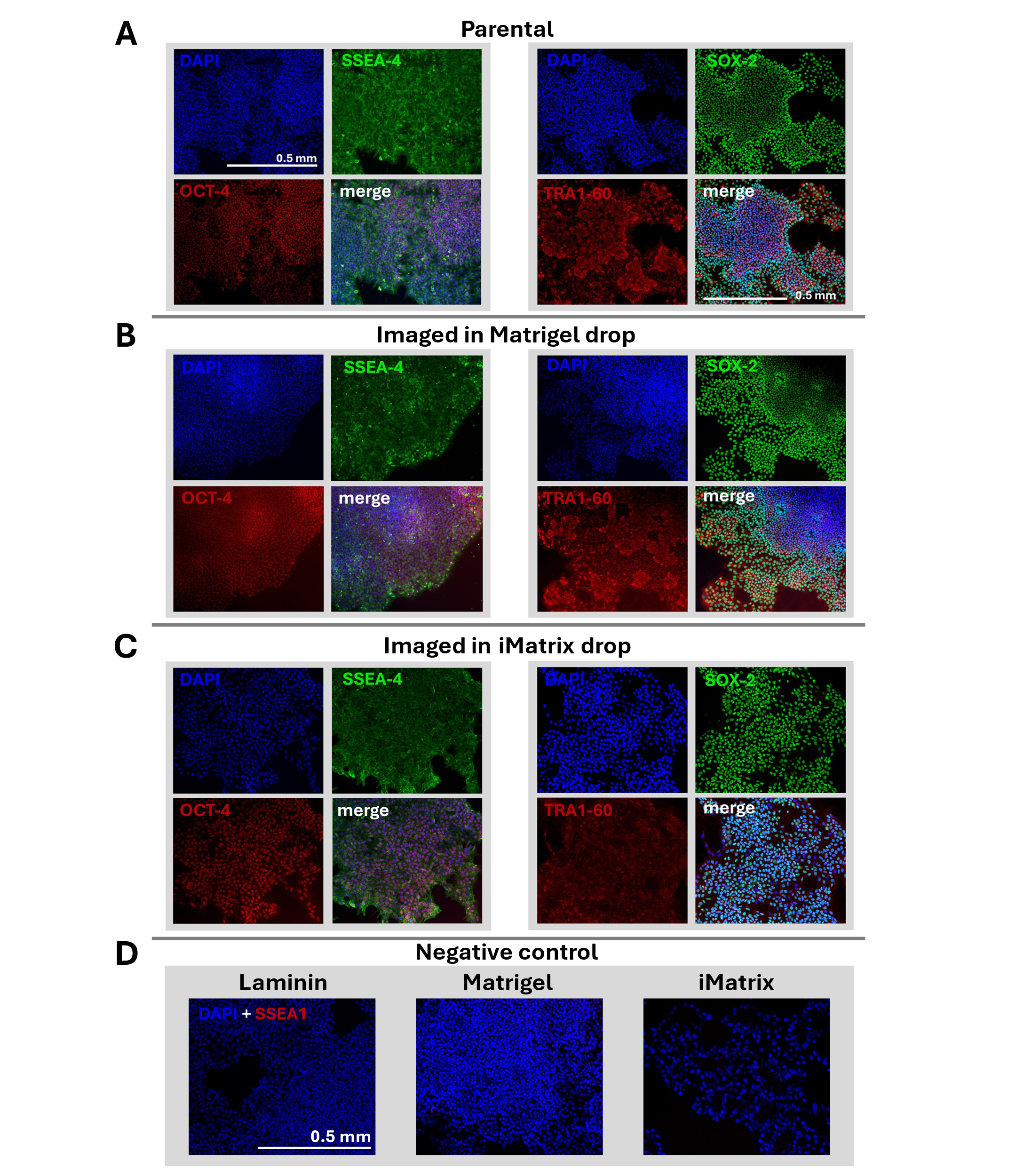
**

**Fig. S4. Immunofluorescence detection of pluripotency markers (SSEA-4/OCT-4 and SOX-2/TRA1-60) in two derived clones grown from single cells imaged in flat drops, and the parental (KOLF2-C1) stem-cell line.**

**(A)** Parental cells.

**(B, C)** Colonies from a single iPSC grown after imaging in a drop of Matrigel or iMatrix. Pluripotency markers continue to be expressed.

**(D)** For each matrix coating, staining for SSEA-1 was used as a negative control.


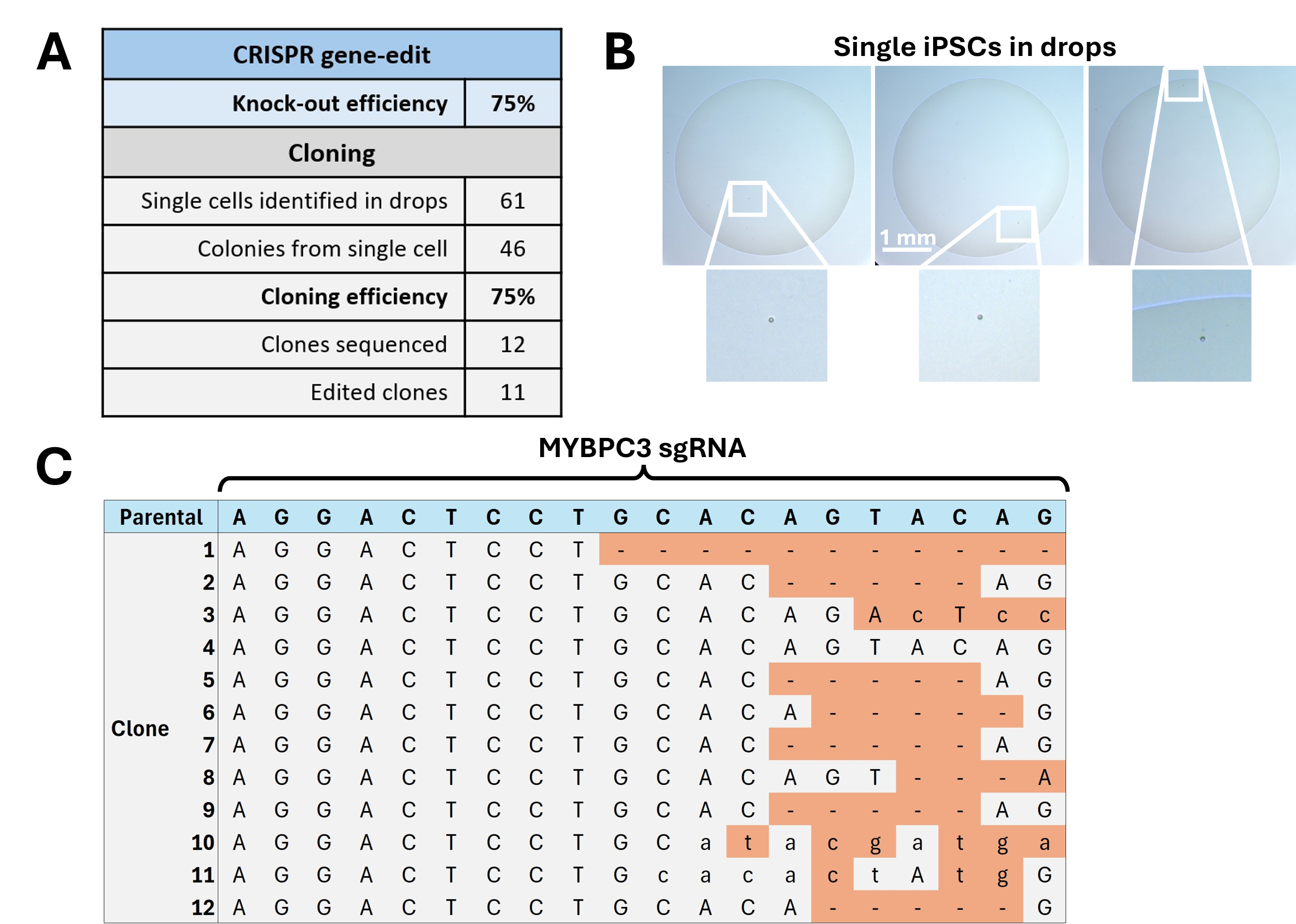
Fig. S5. Integration into a CRISPR gene-edit workflow

Single stem cells were grown on Biolaminin, and cloning efficiency determined on d7.

**(A)** Key figures from our workflow.

**(B)** Example images of single cells in flat drops. Clear drop optics provide an excellent view of a single cell, right up to the drop edge.

**(C)** Target MYBPC3 sequence of the guide RNA (blue background). Twelve clones were selected, and their target DNA amplified and sequenced. Eleven of the 12 contained edits (highlighted in brown).

**
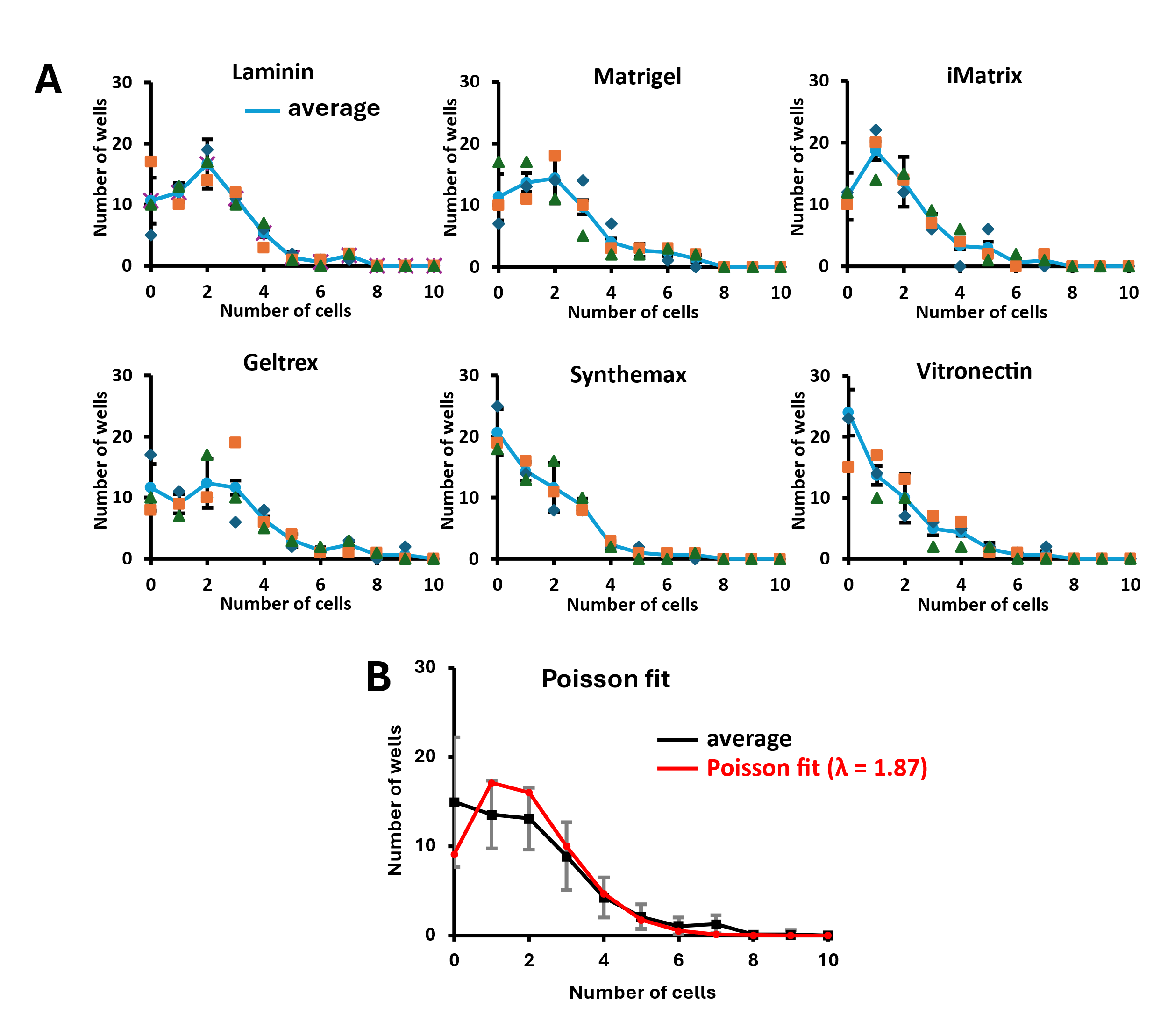
Fig. S6. Cells deposited per well.**

**(A)** Numbers of wells with 0-10 cells for each matrix coating tested. Each graph contains results from three 96-well plates (excluding peripheral wells; different colors show results for different plates).

**(B)** All six plots merged and average plotted (± SD). A maximum likelihood estimation was used to fit the Poisson distribution to the data, giving an estimate of the average number of cells infused into each well.
